# A localized surface plasmon resonance-amplified immunofluorescence biosensor for ultrasensitive and rapid detection of nonstructural protein 1 of Zika virus

**DOI:** 10.1371/journal.pone.0211517

**Published:** 2019-01-31

**Authors:** Kenshin Takemura, Oluwasesan Adegoke, Tetsuro Suzuki, Enoch Y. Park

**Affiliations:** 1 Laboratory of Biotechnology, Department of Bioscience, Graduate School of Science and Technology, Shizuoka University, Suruga-ku, Shizuoka Japan; 2 Laboratory of Biotechnology, Research Institute of Green Science and Technology, Shizuoka University, Suruga-ku, Shizuoka Japan; 3 Department of Infectious Diseases, Hamamatsu University School of Medicine, Handa-yama, Hamamatsu Japan; Brandeis University, UNITED STATES

## Abstract

Among the members of flaviviruses, the Zika virus (ZIKV) remains a potent infectious disease agent, with its associated pandemic prompting the World Health Organization (WHO) to declare it a global public health concern. Thus, rapid and accurate diagnosis of the ZIKV is needed. In this study, we report a new immunofluorescence biosensor for the detection of nonstructural protein 1 (NS1) of the ZIKV, which operates using the localized surface plasmon resonance (LSPR) signal from plasmonic gold nanoparticles (AuNPs) to amplify the fluorescence intensity signal of quantum dots (QDs) within an antigen-antibody detection process. The LSPR signal from the AuNPs was used to amplify the fluorescence intensity of the QDs. For ultrasensitive, rapid, and quantitative detection of NS1 of the ZIKV, four different thiol-capped AuNPs were investigated. Our biosensor could detect the ZIKV in a wide concentration range from 10–10^7^ RNA copies/mL, and we found that the limit of detection (LOD) for the ZIKV followed the order Ab-_L_-cysteine-AuNPs (LOD = 8.2 copies/mL) > Ab-3-mercaptopropionic acid-AuNPs (LOD = 35.0 copies/mL). Immunofluorescence biosensor for NS1 exhibited excellent specificity against other negative control targets and could also detect the ZIKV in human serum.

## Introduction

In the mid-20^th^ century, the causative agent, i.e., the Zika virus (ZIKV), of the vector-borne infectious disease known as Zika fever was discovered in the Zika forest of Uganda [1, 2]. It belongs to the family of genus *Flavivirus* and has a single, positive-stranded RNA genome. The ZIKV shares similar properties with the West Nile virus, Japanese encephalitis, yellow fever and dengue virus [3, 4]. Several outbreaks of the ZIKV have been reported since its discovery, with the most recent being in 2015 in South and North America [5]. The outbreak in Brazil led to the discovery of a direct link between the ZIKV and congenital blindness, microcephaly, and congenital Zika syndrome known as fetal growth restriction [6]. Most recently, a direct association between the ZIKV and Gullain-Barré neurological disorder [7] was reported: it can affect individuals of any age group, thus exposing many population groups to greater risk of infection. Unlike many arboviruses that can spread directly between the host and the vector, the ZIKV is known to spread via body fluids such as semen, saliva, urine and blood [8], thus allowing the virus to spread at an alarmingly rapid rate. Limiting the spread of the virus is problematic because many infected individuals remain asymptomatic [9].

The current ZIKV outbreak has highlighted the main challenges associated with existing diagnostic techniques. Accurate diagnosis of the ZIKV is compounded by the fact that chikungunya and dengue virus yield similar symptoms, such as joint pain, rash and fever [10]. The current serological analysis, which is carried out on body fluids, requires highly skilled technicians to prepare, extract and incorporate samples into advanced analytical instruments [11]. The gold standard viral detection technique, reverse-transcription polymerase chain reaction (RT-PCR), is generally characterized by complex assays, long diagnostic time and expensive peripheral components. Thus, this technique cannot be recommended as a rapid and easy-to-use diagnostic tool for the ZIKV [12]. Furthermore, the enzyme-linked immunosorbent assay (ELISA), which has been used to detect antibodies specific to the ZIKV antigen, suffers from low sensitivity and poor specificity [13]. Additional challenges associated with developing an accurate diagnostic biosensor for the ZIKV are low viral loads, nonspecific binding and cross-reactivity of ZIKV antibodies with other flavivirus antibodies. Therefore, there is an urgent need to develop portable, smart, rapid and accurate detection systems to meet the overwhelming demand for point-of-care treatment of the ZIKV.

Localized surface plasmon resonance (LSPR) biosensors based on fluorescence-enhanced intensity signals have recently emerged as a powerful technique to develop ultrasensitive, rapid and specific detection systems for various biological target analytes [14]. Specifically, our group has adopted this technology to develop biosensors for dengue virus [15] and influenza virus [16, 17]. The concept of the biosensor involves using the LSPR signal from plasmonic nanoparticles (NPs) to mediate the fluorescence intensity signal of semiconductor quantum dot (QD) nanocrystals in a biosensor probe that utilizes molecular beacon or antibodies as receptors. In this work, we report the development of a plasmon-induced immunofluorescence biosensor for NS1 protein of the ZIKV based on an antibody-antigen interaction that is target specific, rapid and ultrasensitive. The NS1 protein (size: ~9 nm) is one of the major antigenic markers for virus infection [18]. The molecule has a size of 42 kD and a homodimer structure. The NS1 protein of ZIKV shows no cross-reactivity with NS1 of other flavivirus species, and it is a very suitable protein as a marker for detection [19]. We selected NS1 of ZIKV as a diagnostic marker because of this point. To enhance the fluorescence signal transducer interface of the QDs upon interaction of the antibody with the virus antigen we investigated four different kinds of thiol-capped gold nanoparticles (AuNPs) as a signal amplifier.

## Materials and methods

### Materials

HEPES buffer, sorbitan monolaurate (Tween 20), polyoxyethylene (20), sulfuric acid sodium acetate, hydrogen peroxide, acetone, chloroform, potassium hydroxide (KOH) and methanol were purchased from Wako Pure Chemical Ind. Ltd. (Osaka, Japan). Oleic acid was purchased from Nacalai Tesque Inc. (Kyoto, Japan). Bovine serum albumin (BSA), HAuCl₄, *N*-hydroxysuccinimide (NHS), *N*-(3-dimethylaminopropyl)-*N*′-ethylcarbodiimide hydrochloride (EDC), cadmium oxide (CdO), 1-octadecene, tellurium (Te), trioctylphosphine oxide (TOPO), hexadecylamine (HDA), selenium (Se), trioctylphosphine (TOP) and sulfur (S) were purchased from Sigma Aldrich co. (St Louis, MO, USA).3-mercaptopropionic acid (3-MPA), thioglycolic acid (TGA), _L_-cysteine (L-cyst) and _L_-glutathione (GSH) as a signal amplifier were also purchased from Sigma-Aldrich co. (St Louis, MO, USA). GSH with molecular weight (MW) of 307.32 is a tripeptide consisting of glutamic acid, cysteine and glycine. There is an amide bond between the amino group of cysteine and a carboxy group on the side chain of glutamic acid. TGA (MW: 92.11) is a kind of thiol ligand that easily oxidizes in air to form disulfide and possesses a terminal carboxylic group that can be used for bonding to external moieties. The acid 3-MPA (MW: 106.14) is a typical thiocarboxylic acid having a carboxyl group and is widely used as a capping. The amino acid _L_-cyst (MW: 121.16) has an amino group and a carboxyl group and is stable under acidic conditions, but it is readily oxidized with air by trace amount of heavy metal ions to become cystine under neutral and alkaline conditions. Goat anti-rabbit IgG-HRP was purchased from Santa Cruz Biotechnology (CA, USA). Tetramethylbenzidine (TMBZ) was purchased from Dojindo (Kumamoto, Japan). Anti-ZIKV NS1 protein antibody was purchased from Gene Tex Inc. (Irvine, CA, USA), which does not react with any other type of flavivirus group of the NS1 protein [20]. Zika virus recombinant NS1 antigen was purchased from the Native Antigen (Oxford, UK). ZIKV strain PRVABC-59 was used in this study. Influenza viruses A/California/07/2009 (H1N1) and A/Netherlands/219/03 (H7N7) were purchased from Vircell Microbiologists (Granada, Spain) and Prospec-Tany TechnoGene Ltd. (Rehovot, Israel), respectively. Norovirus-like particles (NoV-LPs) was prepared in Sf-9 cell culture [21] and used for a selectivity test. All experiments were carried out using high-purity deionized (DI) water (> 18 MΩ·cm).

### Instrumentation

UV/vis absorption and fluorescence emission measurements were performed using a filter-based multimode microplate reader (Infinite F500; TECAN, Ltd, Männedorf, Switzerland). Transmission electron microscopy (TEM) analysis was carried out using a TEM (JEM-2100F; JEOL, Ltd., Tokyo, Japan) operated at 100 kV. Zeta potential and hydrodynamic particle size were measured by dynamic light scattering (DLS) using a Zetasizer Nano series (Malvern Inst. Ltd., Malvern, UK). Powder X-ray diffraction (PXRD) measurement was carried out using a RINT ULTIMA XRD (Rigaku Co., Tokyo, Japan) with a Ni filter and Cu-Kα source. Data were collected over 2theta = 5–60° at a scan rate of 0.01°/step and 10 s/point. Conjugation of the Ab to the NPs was confirmed via an ELISA and read through a plate reader from Bio-Rad (model 680, Hercules, USA). Fourier transform infrared spectroscopy was achieved using the FT/IR-6600 (JASCO, Tokyo, Japan).

### Synthesis of confetto-shaped AuNPs and alloyed QDs

The thiol-capped AuNPs were synthesized using the HEPES buffer according to a previously reported procedure [22]. Briefly, in a 3-necked flask containing a mixture of 4 mL of 1 M HEPES buffer and 36 mL of deionized (DI) water, 1 mL of 20 mM HAuCl_4_ was added. The solution was vigorously stirred and the slow formation of AuNPs was evident by the steady change in the color of the solution to purple. Purification of the AuNPs was carried out by centrifugation; thereafter, the AuNPs were suspended in the DI water. The AuNPs were then functionalized with the respective thiol ligands of MPA, _L_-cyst, TGA and GSH by first adding HCl to the solution and adjusting the pH to 2, and then adding the appropriate volume of thiol solution. The thiol-capped AuNPs were purified by centrifugation and dissolved in 2 mL of ultrapure DI water. The synthesis of the alloyed QDs was carried out according to a previously reported procedure [17].

#### Conjugation of anti-NS1 Ab to AuNPs and CdSeTeS QDs

Carbodiimide EDC/NHS coupling chemistry was used to conjugate the anti-NS1 Ab to the QDs and the respective thiol (MPA, TGA, _L_-cyst and GSH)-capped AuNPs. In a separate reaction vial, the carboxylate groups on the respective AuNPs and QDs (1 mL aqueous solution) were activated with 15 μl of 1 mg/mL EDC and stirred at room temperature for 30 min. Thereafter, 5.1 μg/mL of anti-NS1 Ab was incorporated into each of the systems, and the mixture was stirred for 5 min at 7°C. Then, 15 μl of 1 mg/mL NHS was added to the EDC-activated anti-NS1 Ab-conjugated QDs and the respective AuNPs solutions to stabilize the amide bond formation. Each solution was allowed to stir overnight at 7°C. Purification of the anti-NS1 Ab-conjugated QDs and respective AuNPs was carried out via centrifugation at 3000 g for 10 min, followed by being dissolved in 2 mL of DI water.

#### ELISA for binding confirmation

The binding of the anti-NS1 Abs to the QDs and AuNPs was confirmed using the ELISA. Briefly, 100 μl of conjugated Ab-QDs and Ab-AuNPs were added to separate wells of a 96-well polystyrene plate and incubated at 4°C overnight. To confirm the specificity of the assay, 100 μl of 2% bovine serum albumin (BSA), as a negative control, was added to a separate well. After incubation overnight, the solution was washed three times using a solution of 200 μl PBS buffer containing 5% of Tween (PBST), and a blocking agent (10 μl of 5% skim milk) solution was added to each well and subsequently removed by washing three times with PBST. A dilution solution of 100 μl of anti-rabbit IgG-horseradish with 2% BSA (1:4000) was added to each well and incubated at room temperature for 1 h. A coloring chromogenic agent, 100 μl of TMBZ, was added to each well, which triggered a blue color as a confirmation of binding affinity between the Ab-QDs and Ab-AuNPs. The color of the solution changed from blue to yellow when the reaction was stopped with 50 μl of 10% H_2_SO_4_. The absorbance of the solution at 450 nm was measured using a microplate reader (Infinite F500; TECAN) embedded with a reference filter at 655 nm.

### Fluorescence immunoassay for NS1 of the ZIKV

Anti-NS1 Ab-MPA-AuNPs and anti-NS1 Ab-QDs were mixed at the ratio of 1:3 in a 96-well plate and used to detect each concentration of the ZIKV. The ZIKV solutions (20 μl), in the concentration range of 10–10^7^ RNA copies/mL, were each mixed with 80 μl of the anti-NS1 Ab-MPA-AuNPs and anti-NS1 Ab-QDs detection systems and allowed to incubate for 3 min prior to reading the fluorescence signal. To investigate how thiol capping on the AuNP surface influences the fluorescence intensity signal, anti-NS1 Ab-TGA-AuNPs, anti-NS1 Ab-_L_-cyst-AuNPs and anti-NS1 Ab-GSH-AuNPs were mixed in a separate solution with the anti-NS1 Ab-QDs and used for the ZIKV detection system. Additionally, the sensing performance of the ZIKV detection system was evaluated in a complex medium containing human serum. The detection assay was excited at 450 nm the fluorescence detection was carried out in the range of 460–700 nm. The assay was repeated three times and the average values with standard deviation were shown.

### Detection concept of the biosensor

**Fig 1:**
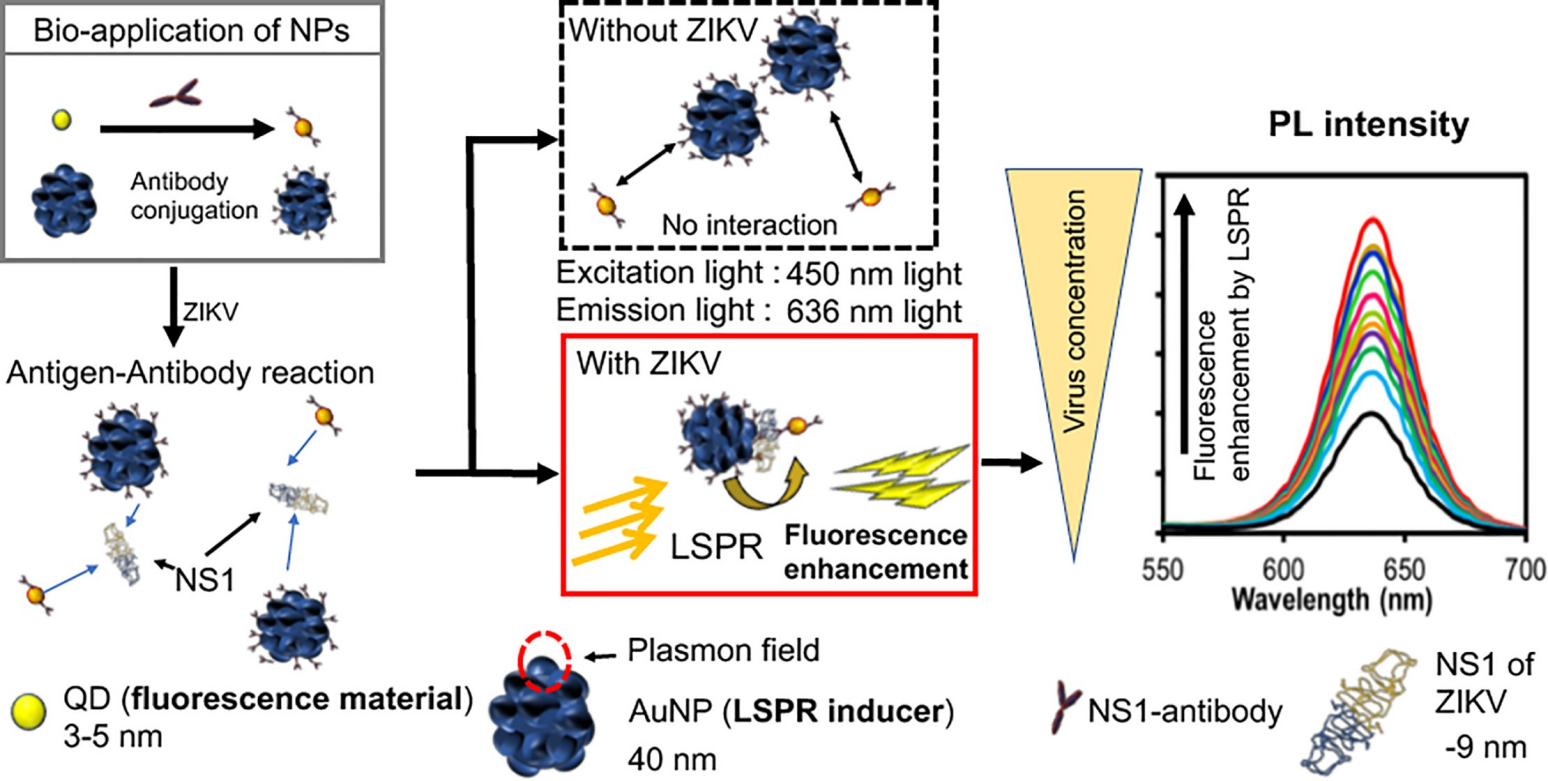
Schematic representation of the LSPR-amplified immunofluorescence biosensor. Anti-NS1 antibody-conjugated AuNPs and QDs are captured ZIKV. LSPR is induced by the close distance of two nanoparticles and enhances the fluorescence intensity.

The detection concept of the biosensor is shown in Fig 1. The AuNPs act as a signal amplifier, while the QDs act as a fluorophore signal transducer. In the presence of the target NS1 of the ZIKV, the Ab on the surface of AuNPs and QDs binds the antigen on the NS1, and the LSPR signal from the AuNPs amplifies the fluorescence intensity of the QDs because the two particles are close.

## Results and discussion

### Characterization

**Fig 2:**
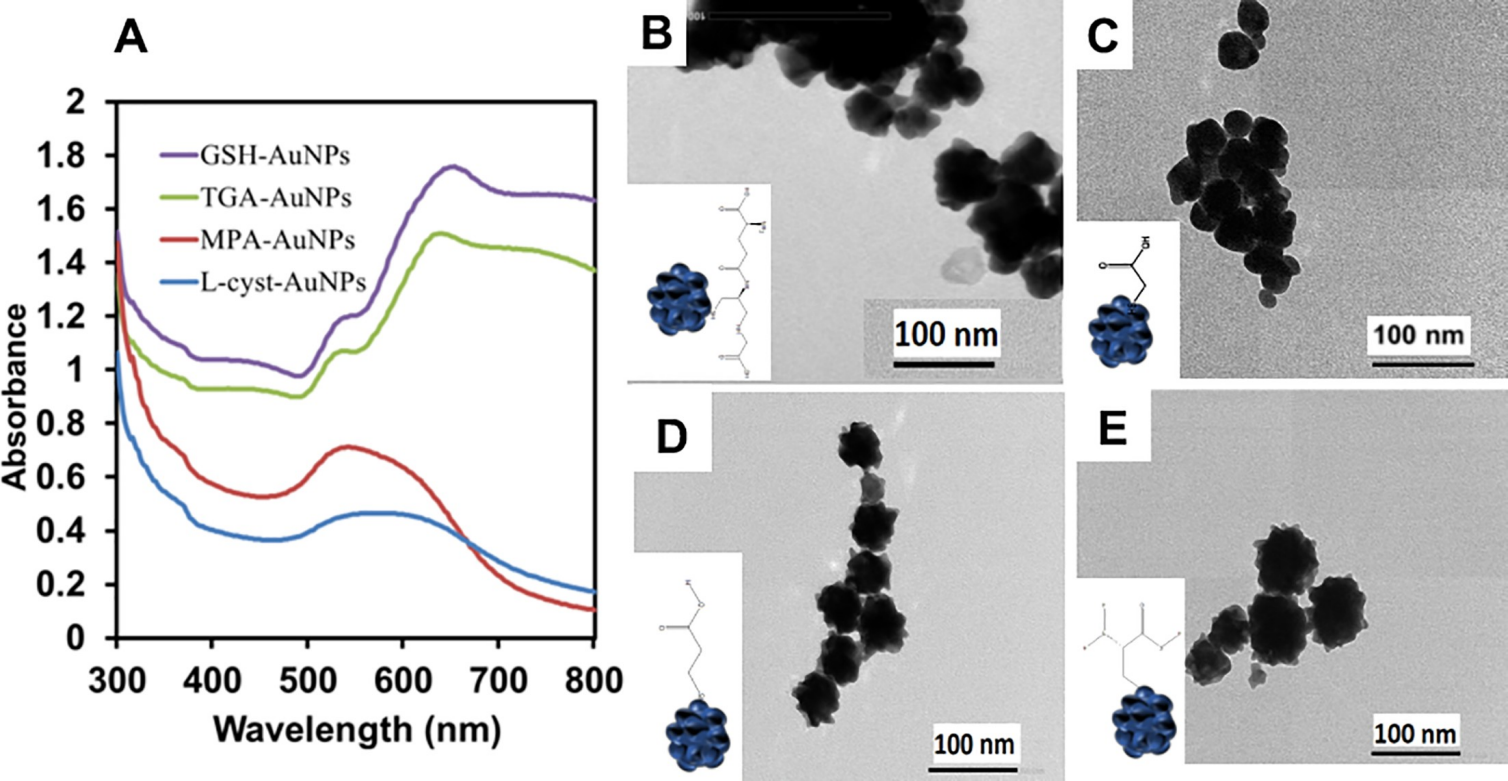
**UV/vis absorption spectra (A) and TEM images (B–E) of the thiol-capped AuNPs.** (B), (C), (D), and (E) denote GSH-, TGA-, MPA-, and _L_-cyst-conjugated AuNPs, respectively.

Four thiol-functionalized AuNPs were synthesized and conjugated to the anti-NS1 Ab. The chemical structures of the four compounds are shown in S1 Fig. The UV/vis absorption spectra of the respective GSH-, TGA-, MPA-, and _L_-cyst-capped AuNPs are shown in Fig 2A. GSH- and TGA-capped AuNPs display surface plasmon resonance (SPR) bands between 494–554 nm and a peak maximum at 654 nm and 634 nm, respectively, while MPA- and _L_-cyst-capped AuNPs are characterized by a broad SPR peak in the range of 526–648 nm. The broad absorption spectra of MPA- and _L_-cyst-capped AuNPs are the characteristic absorption wavelengths of the confetto-shaped AuNPs. On the other hand, GSH- and TGA-capped AuNPs showed a narrow peak, which was typical one of spherical-shaped AuNPs. The spectra on the longer wavelength side is a peak showing aggregation of AuNPs. From the absorbance spectra, changes in the surface shape can be predicted. The corresponding TEM images of the AuNPs reveal their surface morphology (Fig 2B–2E), which is characterized by heterogeneity. Compared to the shape morphology for each of the NPs, the GSH-AuNPs (Fig 2B) exhibit a quasi-spherical morphology, while the MPA- (Fig 2D) and _L_-cyst-capped AuNPs (Fig 2E) are mostly characterized by the confetto-shaped surface morphology.

The hydrodynamic particle size and Zeta potential curves of the thiol-capped AuNPs and the respective Ab-AuNPs are shown in S2 Fig, respectively, while the respective values are listed in Table 1. The sizes of the thiol-capped AuNPs were all less than 100 nm, thus revealing the relatively monodispersed nature of the particles in solution. The increase in hydrodynamic size observed for each of the Ab-AuNPs relative to the unconjugated AuNPs provides direct evidence of the strong binding process between the NPs and the Ab. The percentage increase in hydrodynamic size after binding of the Ab to the AuNPs follows the order of Ab-GSH-AuNPs (62%) >Ab-_L_-cyst-AuNPs (36%) >Ab-TGA-AuNPs (32%) >Ab-MPA-AuNPs (27.6%) (S2A–S2D Fig). The Zeta potential charge of the particle surface of Ab-_L_-cyst-AuNPs, Ab-TGA-AuNPs, and Ab-MPA-AuNPs was shifted more positively in comparison to that of Ab-GSH-AuNPs (S2A1–S2D1 Fig), thus suggesting that the antibody was conjugated to the NP surface with respect to changes in the Zeta potential charge.

**Table 1:** Hydrodynamic sizes and Zeta potential values of the thiol-capped AuNPs and the Ab-AuNPs.

Sample	Hydrodynamic particle size (nm)	Zeta potential (mV)
**GSH-AuNPs**	21.24±8.42	-45.2±9.9
**Ab-GSH-AuNPs**	55.54±19.31	-39.5±7.6
**TGA-AuNPs**	42.37±14.21	-35.4±12.4
**Ab-TGA-AuNPs**	62.73±21.32	-42.7±7.6
**MPA-AuNPs**	40.05±13.69	-40.6±11.1
**Ab-MPA-AuNPs**	55.33±20.94	-29.2±8.2
_**L**_**-cyst-AuNPs**	46.33±16.81	-38.4±11.0
**Ab-**_**L**_**-cyst-AuNPs**	72.82±25.37	-29.4±10.3

The corresponding DLS curves for the unconjugated QDs and the Ab-QDs (S3 Fig) reveals a percentage increase of 69% in hydrodynamic diameter after conjugation to the Ab. The hydrodynamic particle size value determined from the DLS curves was used to assess the aggregation state of the NPs and the Ab-conjugated NPs.

**Fig 3:**
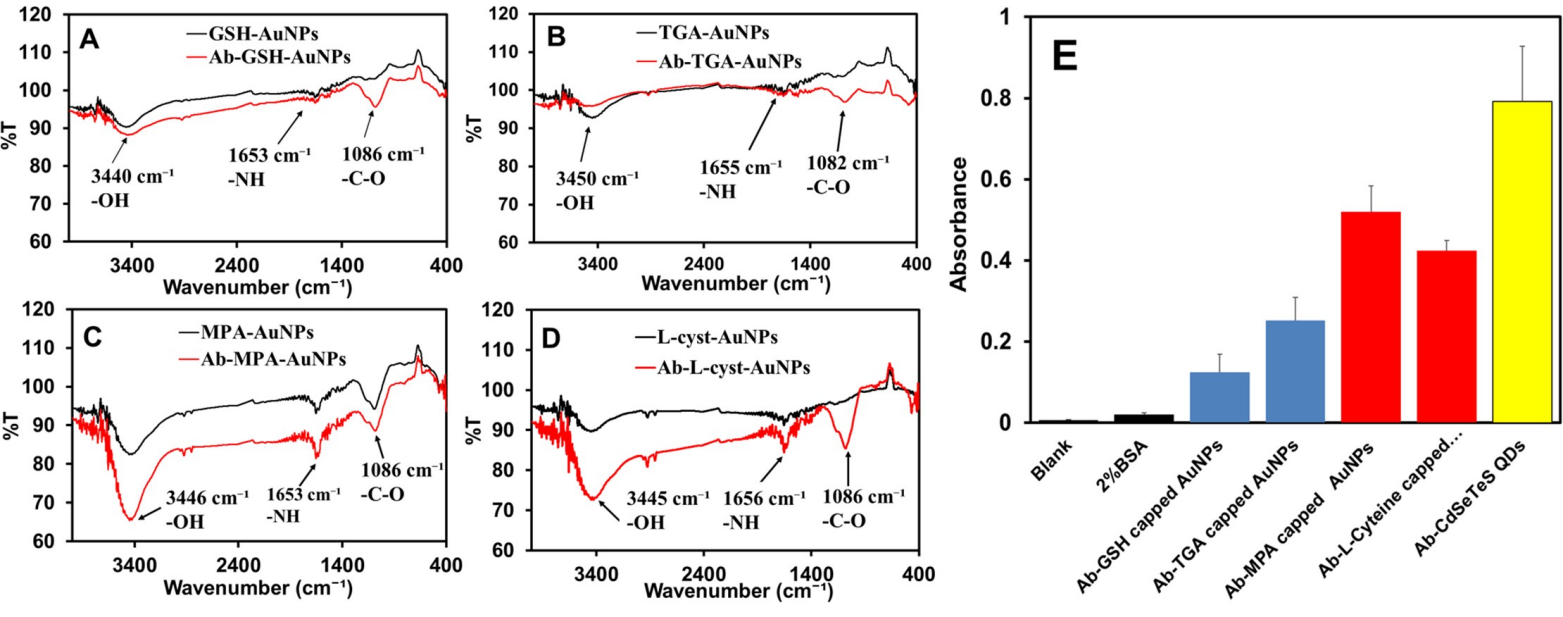
FT-IR spectra of thio-functionalized AuNPs and their binding assay. AuNPs were conjugated by GSH (A), TGA (B), MPA (C), and _L_-cys (D). (E) ELISA showing the strong binding affinity of the Ab to the QDs and AuNPs. The 2% of BSA was used as a negative control.

The FT-IR analysis of the thiol-capped AuNPs and the respective Ab-AuNPs was carried out to confirm the amide bond formation between the amino group on the Ab and the carboxylate group on the thiol-capped AuNPs. It was observed that the intensity of the–OH band and–C = O-NH band for the Ab-AuNPs was stronger for Ab-MPA-AuNPs and Ab-_L_-cyst-AuNPs, than for Ab-GSH-AuNPs and Ab-TGA-AuNPs (Fig 3A–3D), The clear difference in the intensity of the band signal is attributed to the binding affinity of the Ab to the AuNPs. Fig 2E shows the ELISA results confirming the covalent binding between the Ab-QDs and Ab-AuNPs. The strong absorbance for the Ab-QDs, Ab-_L_-cyst-AuNPs and Ab-MPA-AuNPs relative to the negative control (2% BSA) confirms the covalent binding interaction. MPA- and _L_-cyst-AuNPs also show an efficiency of antibody conjugation that is better than that of GSH- and TGA-AuNPs.

### LSPR-amplified detection of NS1 of the ZIKV

**Fig 4:**
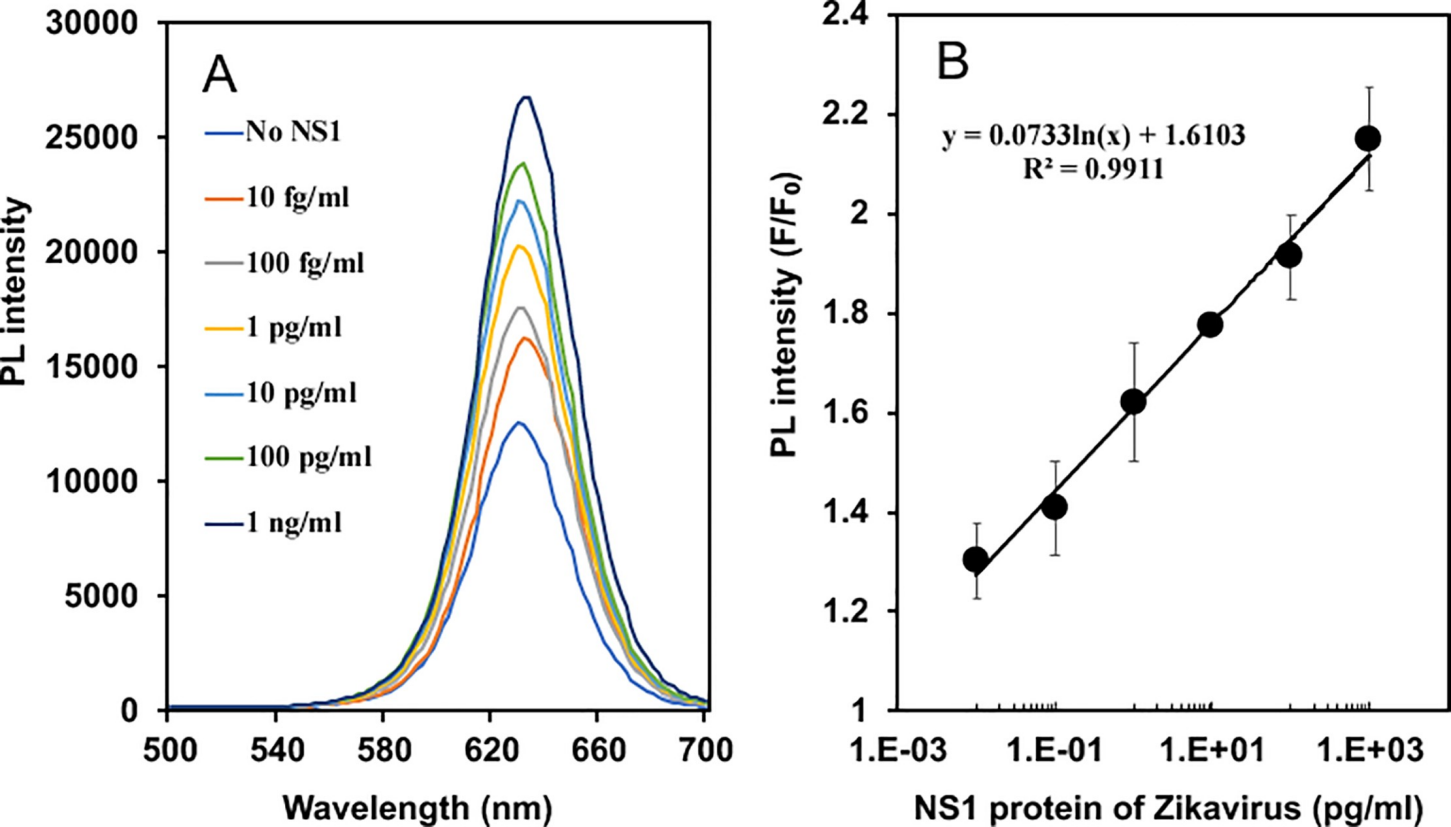
Fluorescence intensity in ZIKV detection using Ab-MPA-AuNPs and Ab-QDs and calibration curve. Fluorescence enhancement spectra (A) of the Ab-QDs and calibration curve (B) of the recombinant NS1 antigen using the LSPR signal amplifier of Ab-MPA-AuNPs.

The ratio of Ab-AuNPs and Ab-QDs were optimized before detection of the ZIKV because the fluorescence signal was affected by the ratio of QD and AuNP. High ratio of Ab-QDs showed increased fluorescence intensity (S4 Fig), which led to increase in the fluorescence baseline and to less enhanced fluorescence intensity. On the other hand, high ratio of Ab-AuNPs decreased the fluorescence intensity (S4 Fig), which also led to decrease the fluorescence baseline and to less enhanced fluorescence intensity. A ratio of Ab-QDs: Ab-NPs = 3: 1 was appropriate for inducing the LSPR signal. Ab-MPA-AuNP was used to detect the NS1 antigen of the ZIKV in the LSPR-amplified biosensor. The fluorescence intensity of the biosensor was dependent on the concentration of the NS1 antigen in the region from 10 pg/mL to 1 ng/mL (Fig 4). The limit of detection (LOD) was evaluated by multiplying the standard deviation of blank measurements (n = 10) by 3 and dividing by the slope of the calibration curve. The LOD was 1.28 fg/mL in DI water, which shows that this sensor can detect NS1-ZIKV with high sensitivity.

ELISA was used to confirm that NS1 was contained in the culture medium infected with the ZIKV (S5 Fig). The immunofluorescence detection of NS1 of the ZIKV was triggered by the LSPR-amplifying effect of the Ab-AuNPs on the fluorescence intensity signal of the Ab-QDs. To confirm that the LSPR signal amplified the fluorescence of the QDs, we carried out the detection of the ZIKV without the LSPR effect from the plasmonic NPs. The poor quantitative detection of NS1 using the immuno-QDs without the LSPR-amplified signal from the plasmonic NPs is shown (S6 Fig). This result demonstrates that the immunofluorescence intensity could not be amplified without the LSPR effect and ensures that NS1 of the ZIKV can be detected quantitatively.

**Fig 5:**
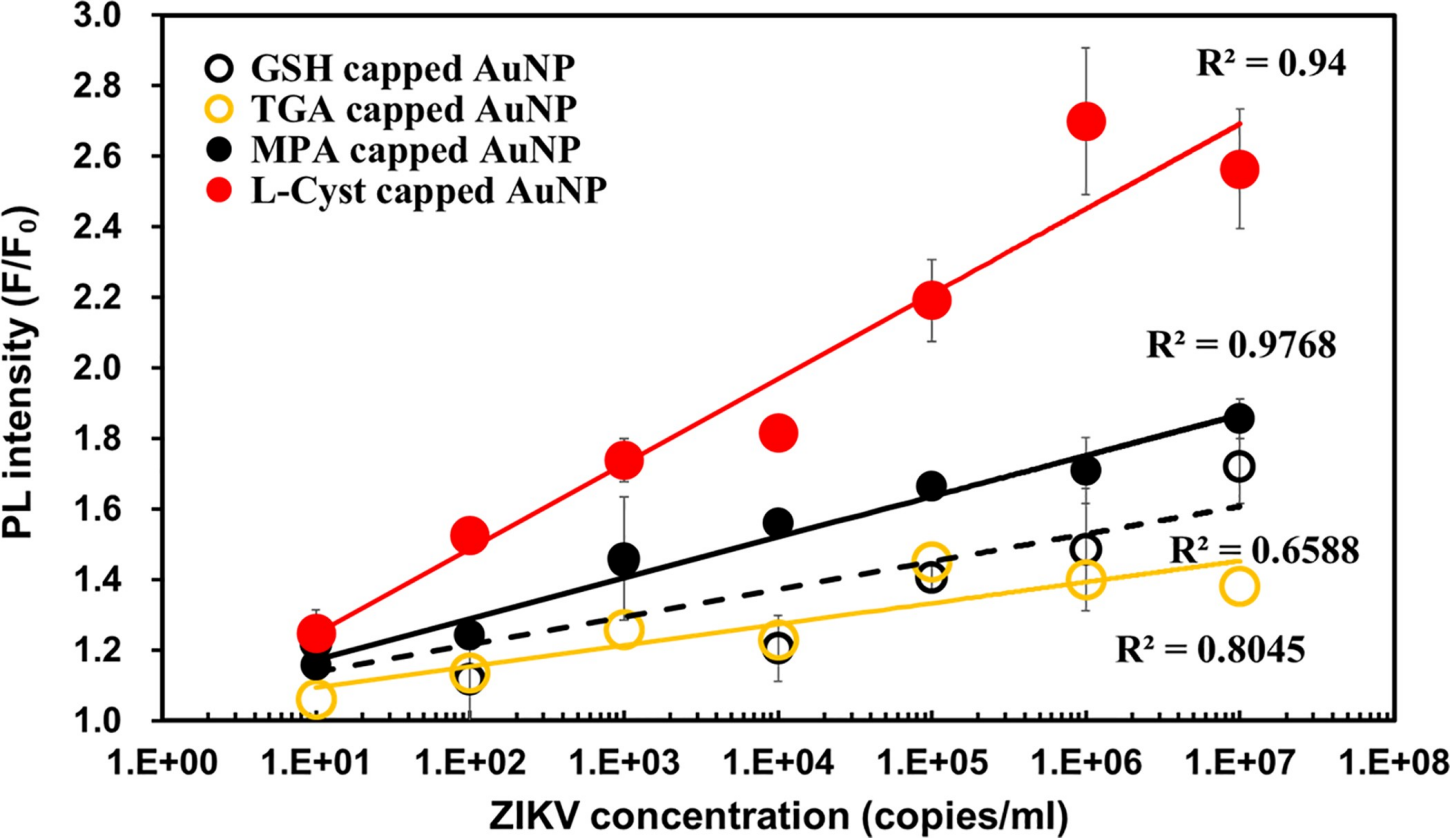
Calibration curves of ZIKV detection using 4 kinds of thiol-functionalized AuNPs. The LSPR signal amplifiers of _L_-cyst-AuNPs (red closed circles) and Ab-MPA-AuNPs (black closed circles) showed higher correlation coefficients than those of Ab-GSH-AuNPs (black open circles) and Ab-TGA-AuNPs (yellow open circles).

The LSPR-amplified fluorescence enhancement spectra for ZIKV detection using the respective Ab-GSH-, Ab-TGA-, Ab-MPA-, and Ab-_L_-cyst-AuNPs are shown in S7 Fig. Ab-TGA- and Ab-GSH-AuNPs, which showed low LSPR-amplified signal and low linearity. Meanwhile, in the cases of Ab-MPA- and Ab-_L_-cyst-AuNPs, the ZIKV was successfully detected in a wide range of concentrations from 10 copies/mL to 10^7^ copies/mL, and with higher sensitivity (Fig 5). The LOD was in the order Ab-_L_-cyst-AuNPs (LOD = 8.2 copies/mL) > Ab-MPA-AuNPs (LOD = 35.0 copies/mL) > Ab-TGA-AuNPs (LOD = 55.8 copies/mL) > Ab-GSH-AuNPs (LOD = 57.9 copies/mL). L-cyst has one carboxyl group and one amino group, but MPA only one carboxyl group (S1 Fig). Here, additional AuNPs may bind to amino group of L-cyst-AuNPs, compared to MPA-AuNPs. Therefore, higher possibility of forming Ab-QD-NS1-Ab-L-cyst-AuNPs sandwich structure than that of Ab-QD-NS1-Ab-MPA-AuNPs, is surmised. This led to more enhanced LSPR effect in L-cyst-capped AuNPs than that in the MPA-AuNPs. Comparison of the quantitative detections revealed that Ab-_L_-cyst-AuNPs amplified the fluorescence intensity of Ab-MPA-AuNPs by 4.3-fold.

**Fig 6:**
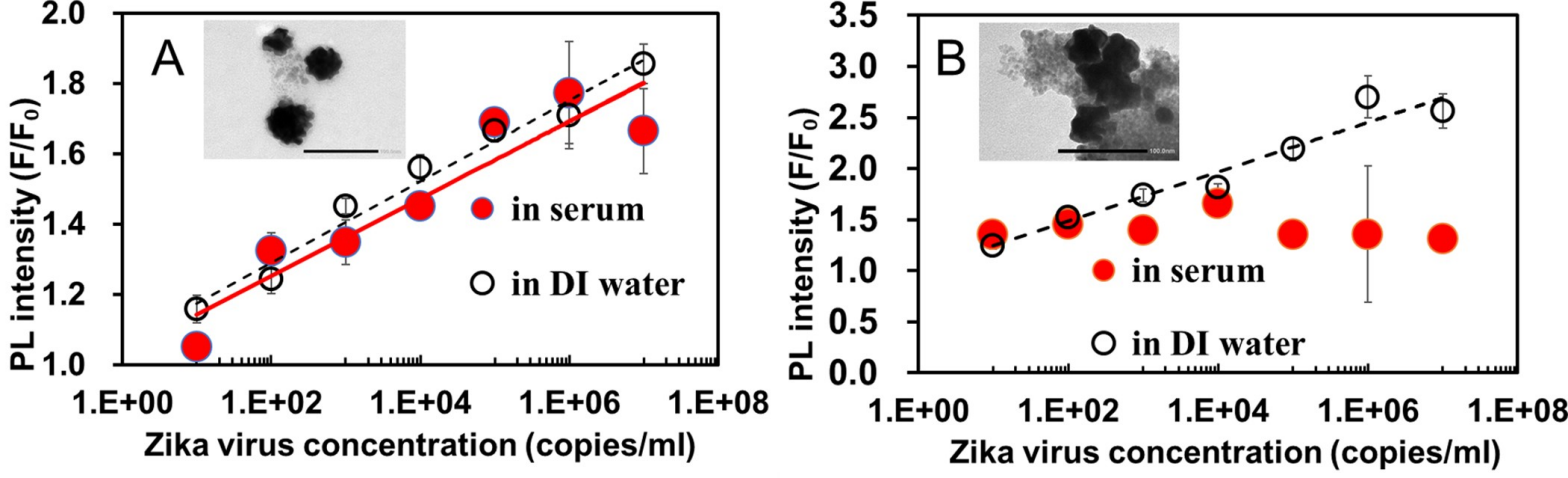
Fluorescence enhancement calibration curve. (A) Ab-MPA-AuNPs and (B) Ab-_L_-cyst-AuNPs were used to detect ZIKV and insets show TEM images of QD-Ab-ZIKV-Ab-MPA-AuNPs and QD-Ab-ZIKV-Ab-_L_-cyst-AuNPs, respectively. Detection media are in human serum (closed red circles) and in DI water (open circles).

The versatility of the LSPR-amplified immunofluorescence biosensor was investigated in a complex biological medium using human serum as a model medium. The linear fluorescence calibration plot of Ab-MPA-AuNPs and Ab-_L_-cyst-AuNPs for NS1 detection is shown in Fig 6A and 6B. The result shows that Ab-MPA-AuNPs amplified the immunofluorescence intensity of the system in human serum better than Ab-_L_-cyst-AuNPs. To better understand the poor amplifying ability exhibited by Ab-_L_-cyst-AuNPs, TEM images of QD-Ab-NS1-Ab-MPA-AuNPs and QD-Ab-NS1-Ab-_L_-cyst-AuNPs in human serum were compared (Fig 6A and 6B). When impure proteins containing serum were added, proteins tend to bind electrostatically with the amino group of L-cyst-AuNPs but are less interacted with MPA-AuNPs because MPA has not amino group. From the TEM image, Ab-_L_-cyst-AuNPs showed a higher level of aggregation in human serum in comparison to Ab-MPA-AuNPs, which can be attributed to the low fluorescence signal response in higher ZIKV concentration of 10^2^ RNA copies/mL.

The hydrodynamic particle sizes of the ZIKV, QD-Ab-NS1-Ab-MPA-AuNPs and QD-Ab-NS1-Ab-_L_-cyst-AuNPs are shown in S8 Fig. The hydrodynamic particle size of the ZIKV was 38.6 nm, and that of Ab-ZIKV-Ab-MPA-AuNPs was 106.8 nm. However, the value for QD-Ab-ZIKV-Ab-_L_-cyst-AuNPs was over 200 nm, which provides direct evidence of the polydisperse nature of the conjugate. Thus, we could conclude that a nonspecific interaction between Ab-_L_-cyst-AuNPs and other proteins in serum could have led to the low sensitivity and aggregation. Functionalization of AuNPs using 3-MPA and L-Cyst, we succeeded in imparting reactivity with antibodies to AuNPs while maintaining the surface structure. Furthermore, by presenting only the carboxyl group by 3-MPA, it was possible to detect NS1 stably even in human serum. In L-Cyst modified AuNPs, the highest fluorescence enhancement effect was found in ultrapure water, but this was due to the fact that the particles were chemically crosslinked by the EDC/NHS reaction and the plasmon field was enhanced and the energy transfer to the QD was occurred more efficiently. This phenomena called coupling effect of AuNPs [23]. However, since the chemical bonding between the nanoparticles increased the particle diameter, when nano-sized ZIKV was present in human serum, the antigen-antibody reaction could not be normally induced, and the fluorescence enhancement effect decreases.

The applicability of the immunofluorescence biosensor system was applied to detect the influenza virus, as shown in S9 Fig. The influenza virus was detected with high sensitivity in the range of 1 fg/mL to 100 pg/mL and in human serum. The results reflect the robustness of our LSPR immunofluorescence biosensor system to detect other target viruses.

The sensitivity of our detection system was compared with other detection methods (Table 2). The comparison shows that the sensitivity of the LSPR immunofluorescence biosensor was comparable to that of the molecular beacon system and was higher than that of the RT-PCR and RT-LAMP methods. Additionally, our detection method has the advantage that DNA extraction and a washing process are not required, which is an attractive property for rapid and onsite ZIKV detection.

**Table 2:** Comparison of detection methods in terms of ZIKV detection.

Detection technique	Signal type	LOD	Reference
**Molecular Beacon**	Fluorescence enhancement	1.7 copies/ml	[24]
**Nanoparticle-enhanced electrical detection**	Electrical signal	10 virus particle/μl	[25]
**RT-LAMP Molecular detection**	Colorimetric	5 pfu/ml	[26]
		1 copy/μl	[27]
**rRT-PCR** **(real-time RT-PCR)**	Polymerase chain reaction	32 genome (0.05 pfu/ml)	[28]
		530 aM (3.2×10^5^ copies/mL)	[29]
		~ 1 fM	[8]
		10^3^ GCE/mL genome copy equivalents	[30]
**LSPR-amplified immunofluorescence**	Fluorescence enhancement	NS1 antigen 1.28 fg/ml (in DI water) 8.2 RNA copies/ml (in DI water) ~ 100 RNA copies/ml (in serum)	This work

#### Specificity of the LSPR-amplified immunofluorescence biosensor

**Fig 7:**
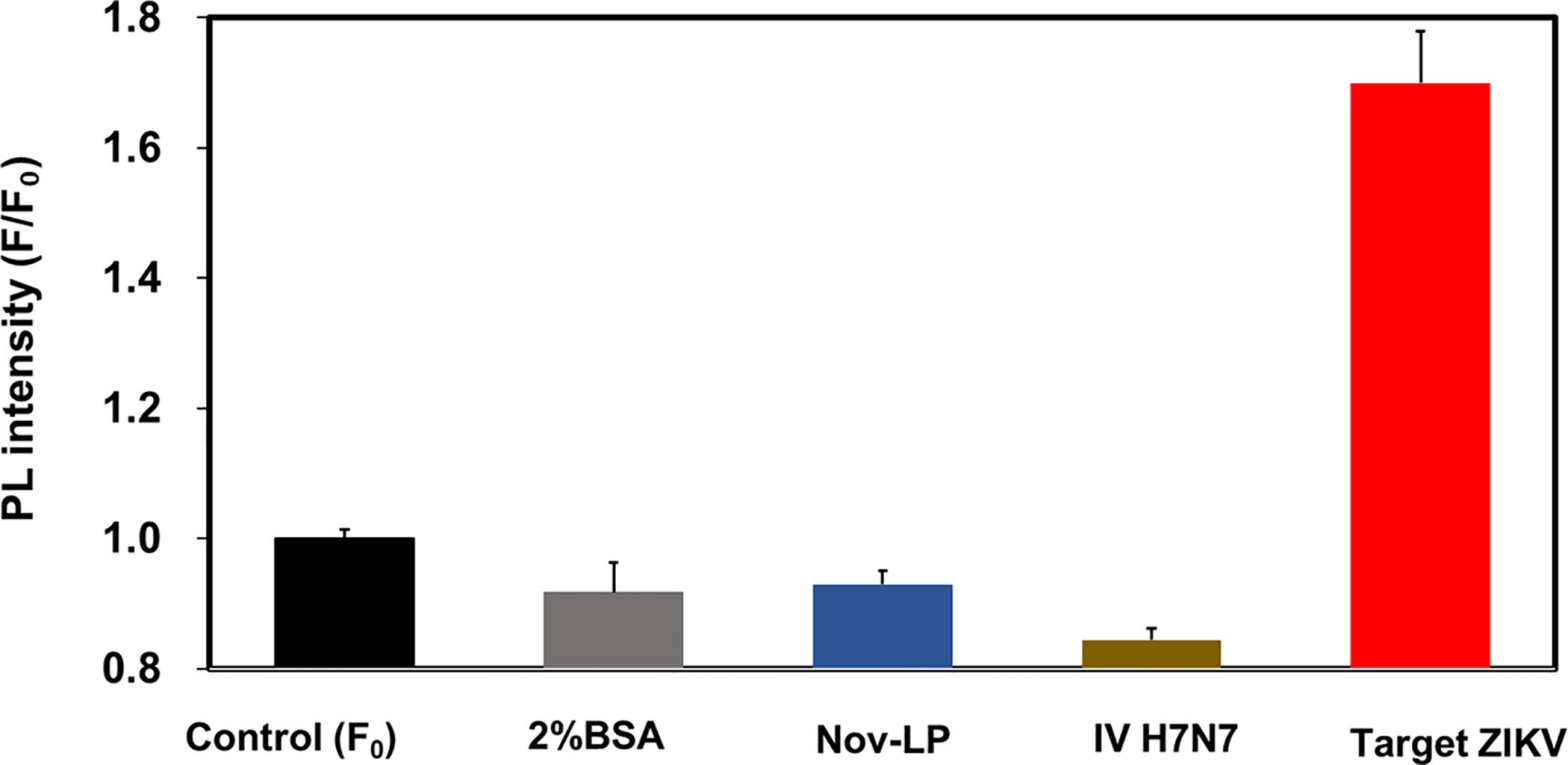
Specificity of our proposed detection method. Fluorescence intensity of ZIKV was compared with those of NoV-LPs, influenza virus A (H7N7) BSA, and BSA as negative control. The concentration of the sample used was 100 ng/mL for NoV-LPs and influenza virus A (H7N7) and 1×10^5^ RNA copies/mL for the ZIKV.

The specificity of the LSPR-amplified immunofluorescence biosensor to detect the ZIKV was investigated in the presence of other negative analytes. BSA, norovirus-like particles (NoV-LP) and influenza virus A (H7N7) were used as a negative control. As shown in Fig 7, none of the tested negative control analytes altered the fluorescence intensity of the biosensor probe. Thus, the results provide a strong indication that the LSPR-induced immunofluorescence biosensor developed in this work is specific for the ZIKV.

## Conclusions

A new LSPR-amplified immunofluorescence biosensor for NS1 of the ZIKV has been developed with characteristic features of rapidity, ultrasensitivity and specificity for ZIKV detection. Four Ab-conjugated thiol-capped AuNPs were investigated for their use as LSPR signal amplifiers within an antigen-antibody detection system, while the Ab-conjugated QD was used as a fluorescent signal transducer. Our results show that NS1 can be detected within a wide range of concentrations from 10 fg/mL to 1 ng/mL with an LOD of 1.28 fg/mL. Based on this result the ZIKV was detected from 10–107 RNA copies/mL with high sensitivity, rapidity and specificity using Ab-MPA-AuNPs. We have also demonstrated that Ab-MPA-AuNPs stably detected the ZIKV in a complex biological medium without any complicated processes.

## Supporting information

S1 Fig Chemical structure of (A) _L_-glutathione (GSH), (B) thioglycolic acid (TGA), (C) 3-mercaptopropionic acid (MPA) and (D) _L_-cysteine.
(TIF)

S2 Fig **DLS hydrodynamic curves (A–D) and Zeta potential (ZP) (A1–D1) for the thiol-capped AuNPs (red curves) and the Ab-AuNPs (green curves).** The thiol capping agents are (A, A1) GSH, (B, B1) TGA, (C, C1) MPA and (D, D1) _L_-cyst. Green and red curves indicate for the thiol-capped AuNPs and the Ab-AuNPs, respectively. The thiol capping are (A1) GSH, (B1) TGA, (C1) MPA and (D1) L-cyst.
(TIF)

S3 Fig DLS hydrodynamic curves for the QDs before (red curve) and after (green curve) conjugation to the antibody. (TIF)

S4 Fig Effect of Ab-QDs and Ab-AuNPs ratio in detection solution. ZIKV concentration was 10 RNA copies/mL.
(TIF)

S5 Fig ELISA showing the strong binding affinity of the NS1 antibody to the NS1 protein in Zika virus sample solution. (TIF)

S6 Fig Fluorescence intensity changes for ZIKV using the immuno-QDs without LSPR-amplified signal from the plasmonic AuNPs. (TIF)

S7 Fig Fluorescence enhancement spectrum of the Ab-QDs as it correlates to the detected ZIKV using the LSPR signal amplifier of A) Ab-GSH-AuNPs, B) Ab-TGA-AuNPs, C) Ab-MPA-AuNPs, and D) Ab- L-cyst-AuNPs.
(TIF)

S8 Fig **DLS hydrodynamic curves for A) QD-Ab-ZIKV-Ab-MPA-AuNPs and B) QD-Ab-ZIKV-Ab-L-cyst-AuNPs.** Peaks of A, B and C are 38.6, 106.8 and 212.0 nm, respectively.
(TIF)

S9 Fig Fluorescence enhancement spectrum of the Ab-QDs. Influenza virus A (H1N1) was detected using the LSPR signal amplifier of Ab-MPA-AuNP in DI water (A), in human serum (B). Calibration curve (C) for Ab-MPA AuNP in DI water (〇) and in human serum (●).
(TIF)

## References

[1] Afsahi S, Lerner MB, Goldstein JM, Lee J, Tang X, Bagarozzi DA Jr, et al Novel graphene-based biosensor for early detection of Zika virus infection. Biosens Bioelectron. 2018;100:85–8. 10.1016/j.bios.2017.08.051 28865242

[2] Gebre Y, Forbes N, Gebre T. Zika virus infection, transmission, associated neurological disorders and birth abnormalities: A review of progress in research, priorities and knowledge gaps. Asian Pac J Biomed. 6(10):815–24.

[3] Kuno G, Chang G-JJ, Tsuchiya KR, Karabatsos N, Cropp CB. Phylogeny of the genus Flavivirus. J Virol. 1998;72(1):73–83. 9420202 10.1128/jvi.72.1.73-83.1998 PMC109351

[4] Sikka V, Chattu VK, Popli RK, Galwankar SC, Kelkar D, Sawicki SG, et al The emergence of Zika virus as a global health security threat: a review and a consensus statement of the INDUSEM Joint Working Group (JWG). J Glob Infect Dis. 2016;8(1):3 10.4103/0974-777X.176140 27013839 PMC4785754

[5] Petersen LR, Jamieson DJ, Powers AM, Honein MA. Zika virus. N Engl J Med. 2016;374(16):1552–63. 10.1056/NEJMra1602113 27028561

[6] Coyne CB, Lazear HM. Zika virus—reigniting the TORCH. Nat Rev Microbiol. 2016;14(11):707 10.1038/nrmicro.2016.125 27573577

[7] Cao-Lormeau V-M, Blake A, Mons S, Lastère S, Roche C, Vanhomwegen J, et al Guillain-Barré Syndrome outbreak associated with Zika virus infection in French Polynesia: a case-control study. The Lancet. 2016;387(10027):1531–9. 10.1016/S0140-6736(16)00562-6 PMC5444521 26948433

[8] Barzon L, Pacenti M, Franchin E, Lavezzo E, Trevisan M, Sgarabotto D, et al Infection dynamics in a traveller with persistent shedding of Zika virus RNA in semen for six months after returning from Haiti to Italy, January 2016. Eurosurveillance. 2016;21(32). 10.2807/1560-7917.ES.2016.21.32.30316 PMC4998504 27542178

[9] Weaver SC, Costa F, Garcia-Blanco MA, Ko AI, Ribeiro GS, Saade G, et al Zika virus: History, emergence, biology, and prospects for control. Antiviral Res. 2016;130:69–80. 10.1016/j.antiviral.2016.03.010 26996139 PMC4851879

[10] Priye A, Bird SW, Light YK, Ball CS, Negrete OA, Meagher RJ. A smartphone-based diagnostic platform for rapid detection of Zika, chikungunya, and dengue viruses. Sci Rep. 2017;7:44778 10.1038/srep44778 28317856 PMC5357913

[11] Shukla S, Hong S-Y, Chung SH, Kim M. Rapid detection strategies for the global threat of Zika virus: current state, new hypotheses, and limitations. Front Microbiol. 2016;7:1685 10.3389/fmicb.2016.01685 27822207 PMC5075579

[12] Kaushik A, Tiwari S, Jayant RD, Vashist A, Nikkhah-Moshaie R, El-Hage N, et al Electrochemical biosensors for early stage Zika diagnostics. Trends Biotechnol. 2017;35(4):308–17. 10.1016/j.tibtech.2016.10.001 28277248 PMC5366270

[13] Vorou R. Letter to the editor: diagnostic challenges to be considered regarding Zika virus in the context of the presence of the vector Aedes albopictus in Europe. Eurosurveillance. 2016;21(10):30161 10.2807/1560-7917.ES.2016.21.10.30161 26988027

[14] Lee J, Takemura K, Park E. Plasmonic nanomaterial-based optical biosensing platforms for virus detection. Sensors. 2017;17(10):2332. 10.3390/s17102332 PMC5677418 29027923

[15] Adegoke O, Park EY. Bright luminescent optically engineered core/alloyed shell quantum dots: an ultrasensitive signal transducer for dengue virus RNA via localized surface plasmon resonance-induced hairpin hybridization. J Mater Chem B. 2017;5(16):3047–58. 10.1039/c7tb00388a 32263996

[16] Lee J, Ahmed SR, Oh S, Kim J, Suzuki T, Parmar K, et al A plasmon-assisted fluoro-immunoassay using gold nanoparticle-decorated carbon nanotubes for monitoring the influenza virus. Biosens Bioelectron. 2015;64:311–7. 10.1016/j.bios.2014.09.021 25240957

[17] Takemura K, Adegoke O, Takahashi N, Kato T, Li T-C, Kitamoto N, et al Versatility of a localized surface plasmon resonance-based gold nanoparticle-alloyed quantum dot nanobiosensor for immunofluorescence detection of viruses. Biosens Bioelectron. 2017;89:998–1005. 10.1016/j.bios.2016.10.045 27825520

[18] Song H, Qi J, Haywood J, Shi Y, Gao GF. Zika virus NS1 structure reveals diversity of electrostatic surfaces among flaviviruses. Nat Struct Mol Biol. 2016;23(5):456 10.1038/nsmb.3213 27088990

[19] Brown WC, Akey DL, Konwerski JR, Tarrasch JT, Skiniotis G, Kuhn RJ, et al Extended surface for membrane association in Zika virus NS1 structure. Nat Struct Mol Biol. 2016;23(9):865 10.1038/nsmb.3268 27455458 PMC5951387

[20] Bollweg BC, Silva‐Flannery L, Spivey P, Hale GL. Optimization of commercially available Zika virus antibodies for use in a laboratory‐developed immunohistochemical assay. J Pathol Clin Res. 2018;4(1):19–25. 10.1002/cjp2.84 29416874 PMC5783976

[21] Ahmed SR, Takemeura K, Li T-C, Kitamoto N, Tanaka T, Suzuki T, et al Size-controlled preparation of peroxidase-like graphene-gold nanoparticle hybrids for the visible detection of norovirus-like particles. Biosens Bioelectron. 2017;87:558–65. 10.1016/j.bios.2016.08.101 27611475

[22] Chen R, Wu J, Li H, Cheng G, Lu Z, Che C-M. Fabrication of gold nanoparticles with different morphologies in HEPES buffer. Rare Metals. 29(2):180–6.

[23] Ghosh SK, Pal T. Interparticle coupling effect on the surface plasmon resonance of gold nanoparticles: from theory to applications. Chem Rev. 2007;107(11):4797–862. 10.1021/cr0680282 17999554

[24] Adegoke O, Morita M, Kato T, Ito M, Suzuki T, Park EY. Localized surface plasmon resonance-mediated fluorescence signals in plasmonic nanoparticle-quantum dot hybrids for ultrasensitive Zika virus RNA detection via hairpin hybridization assays. Biosens Bioelectron. 2017;94:513–22. 10.1016/j.bios.2017.03.046 28343104

[25] Draz MS, Venkataramani M, Lakshminarayanan H, Saygili E, Moazeni M, Vasan A, et al Nanoparticle-enhanced electrical detection of Zika virus on paper microchips. Nanoscale. 2018. 10.1039/c8nr01646a PMC6033661 29881853

[26] Song J, Mauk MG, Hackett BA, Cherry S, Bau HH, Liu C. Instrument-free point-of-care molecular detection of Zika virus. Anal Chem. 2016;88(14):7289–94. 10.1021/acs.analchem.6b01632 27306491 PMC4955015

[27] Kaarj K, Akarapipad P, Yoon J-Y. Simpler, Faster, and Sensitive Zika Virus Assay Using Smartphone Detection of Loop-mediated Isothermal Amplification on Paper Microfluidic Chips. Sci Rep. 2018;8(1):12438 10.1038/s41598-018-30797-9 30127503 PMC6102244

[28] Faye O, Faye O, Diallo D, Diallo M, Weidmann M. Quantitative real-time PCR detection of Zika virus and evaluation with field-caught mosquitoes. Virol J. 2013;10(1):311. 24148652 10.1186/1743-422X-10-311 PMC4016539

[29] Musso D, Nhan T, Robin E, Roche C, Bierlaire D, Zisou K, et al Potential for Zika virus transmission through blood transfusion demonstrated during an outbreak in French Polynesia, November 2013 to February 2014. Eurosurveillance. 2014;19(14):20761 24739982 10.2807/1560-7917.es2014.19.14.20761

[30] Santiago GA, Vázquez J, Courtney S, Matías KY, Andersen LE, Colón C, et al Performance of the Trioplex real-time RT-PCR assay for detection of Zika, dengue, and chikungunya viruses. Nat Commun. 2018;9(1):1391 10.1038/s41467-018-03772-1 29643334 PMC5895813

