# Supplementary figures and images for: A localized surface plasmon resonance-amplified immunofluorescence biosensor for ultrasensitive and rapid detection of nonstructural protein 1 of Zika virus
Source: PLoS One. 2019 Jan 31;14(1):e0211517. doi: 10.1371/journal.pone.0211517 (PMC6355018; doi:10.1371/journal.pone.0211517)

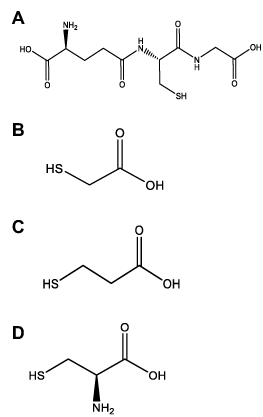

Supplement: S1 Fig — Chemical structure of (A) L-glutathione (GSH), (B) thioglycolic acid (TGA), (C) 3-mercaptopropionic acid (MPA) and (D) L-cysteine. (TIF) [file pone.0211517.s001.tif]

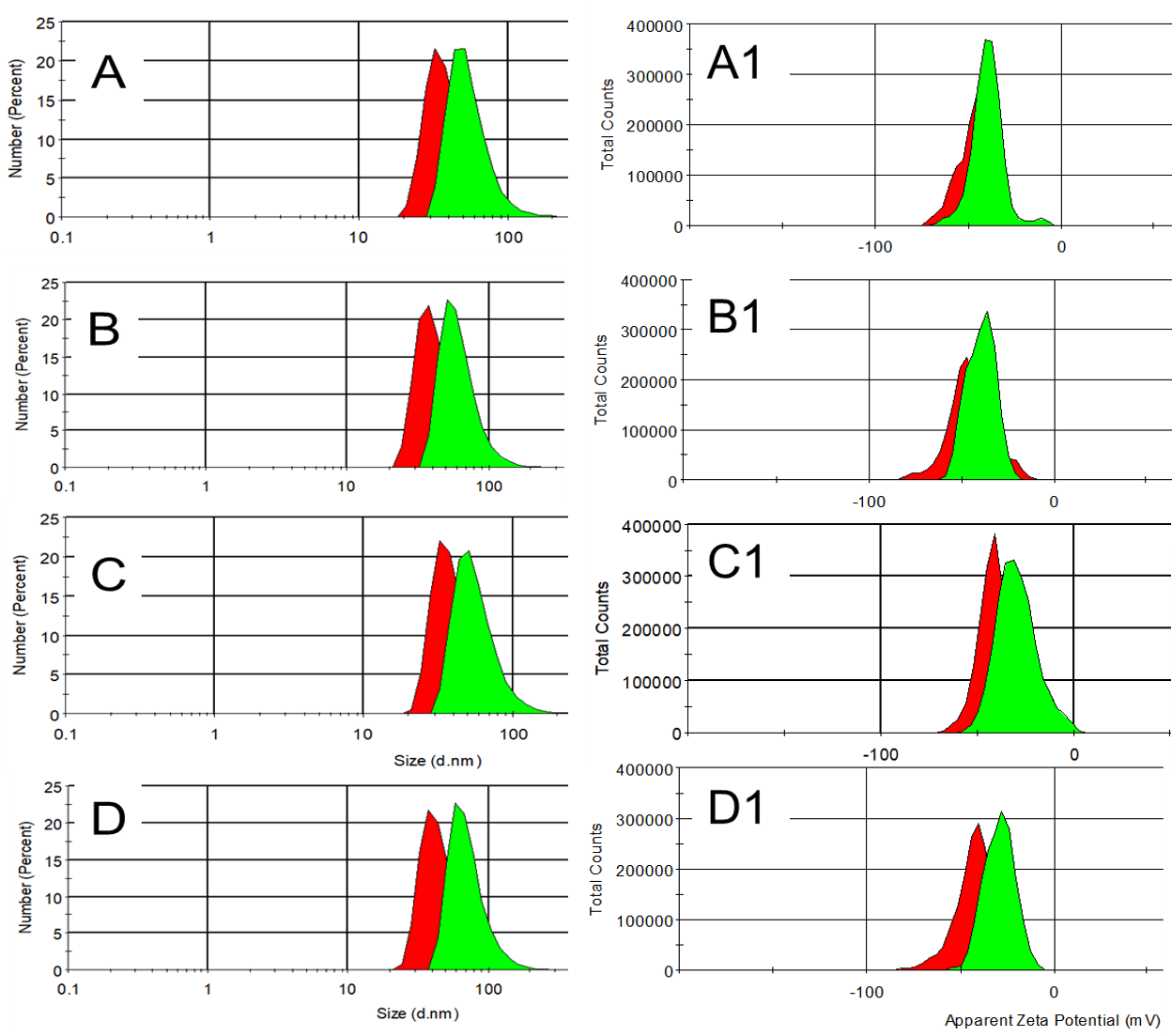

Supplement: S2 Fig — DLS hydrodynamic curves (A–D) and Zeta potential (ZP) (A1–D1) for the thiol-capped AuNPs (red curves) and the Ab-AuNPs (green curves). The thiol capping agents are (A, A1) GSH, (B, B1) TGA, (C, C1) MPA and (D, D1) L-cyst. Green and red curves indicate for the thiol-capped AuNPs and the Ab-AuNPs, respectively. The thiol capping are (A1) GSH, (B1) TGA, (C1) MPA and (D1) L-cyst. (TIF) [file pone.0211517.s002.tif]

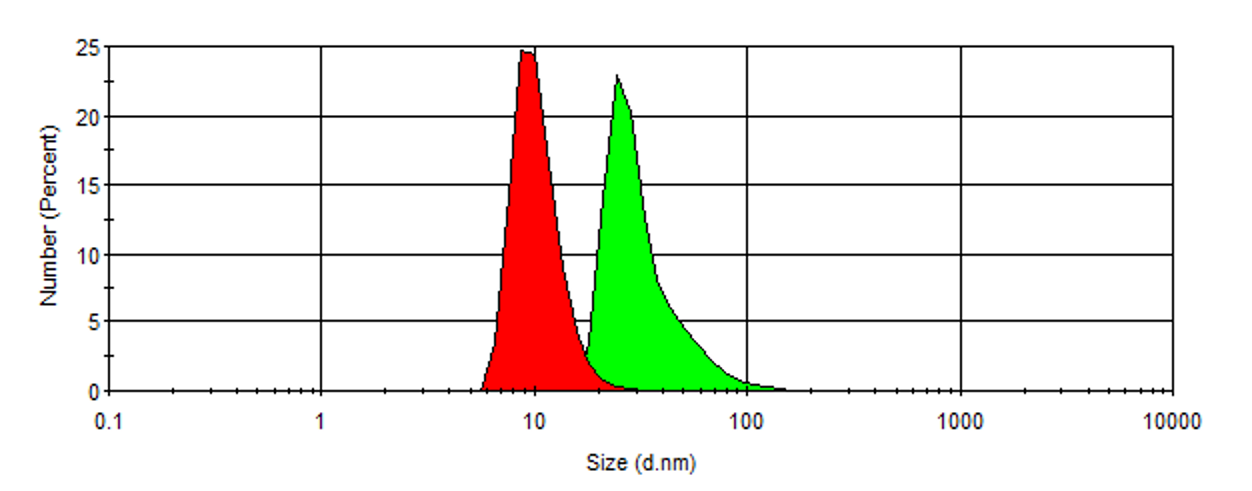

Supplement: S3 Fig — (TIF) [file pone.0211517.s003.tif]

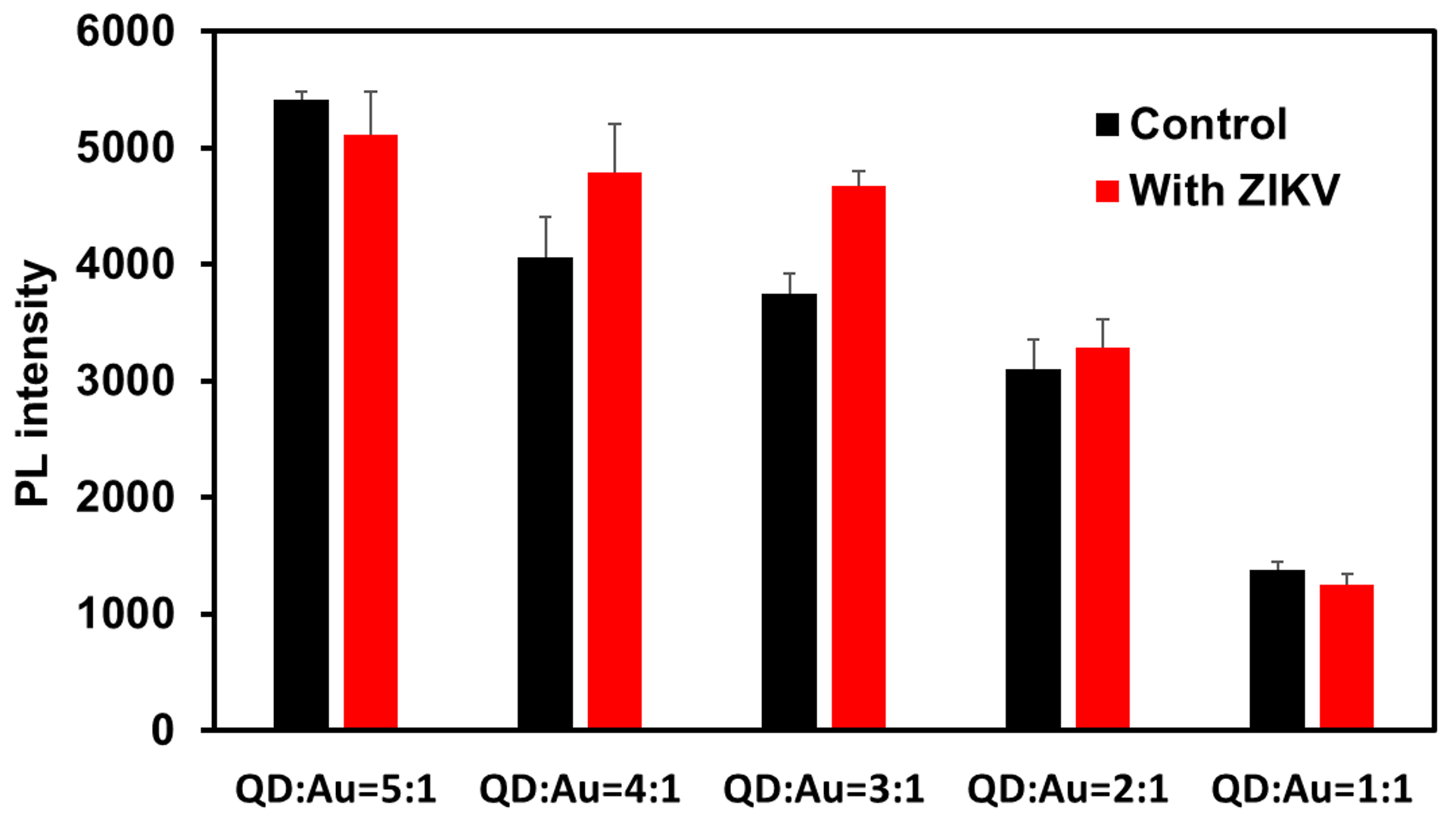

Supplement: S4 Fig — ZIKV concentration was 10 RNA copies/mL. (TIF) [file pone.0211517.s004.tif]

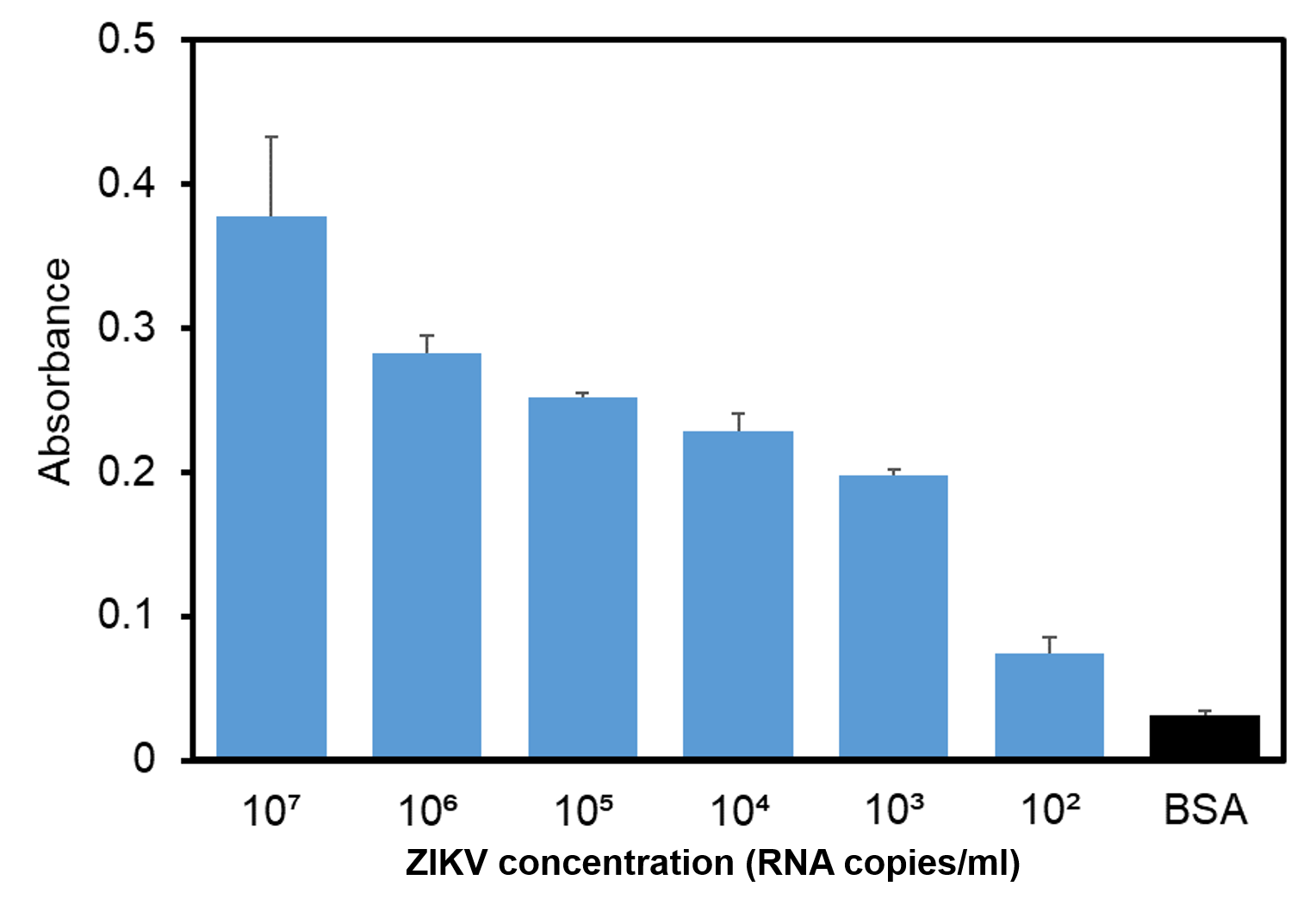

Supplement: S5 Fig — (TIF) [file pone.0211517.s005.tif]

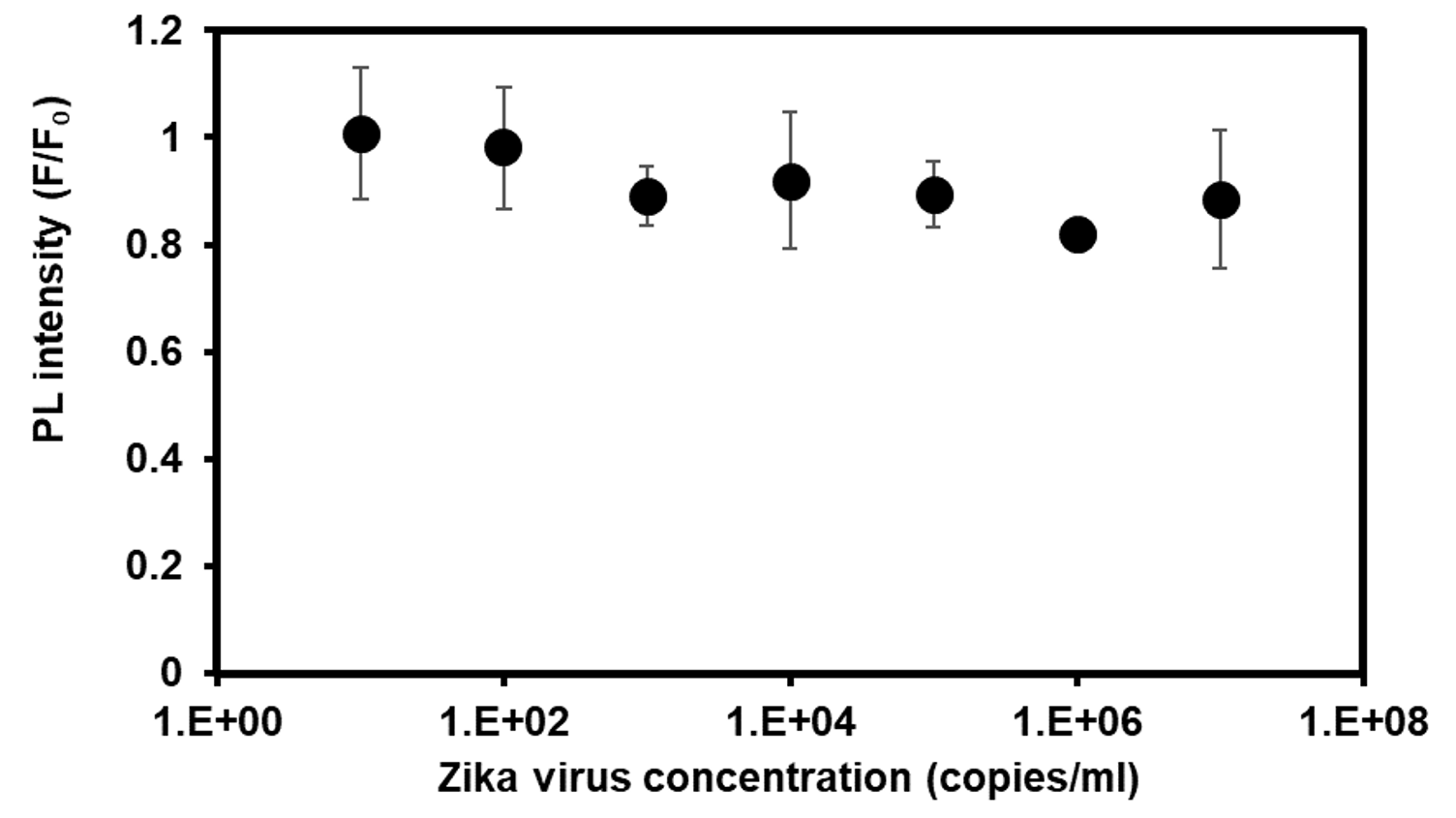

Supplement: S6 Fig — (TIF) [file pone.0211517.s006.tif]

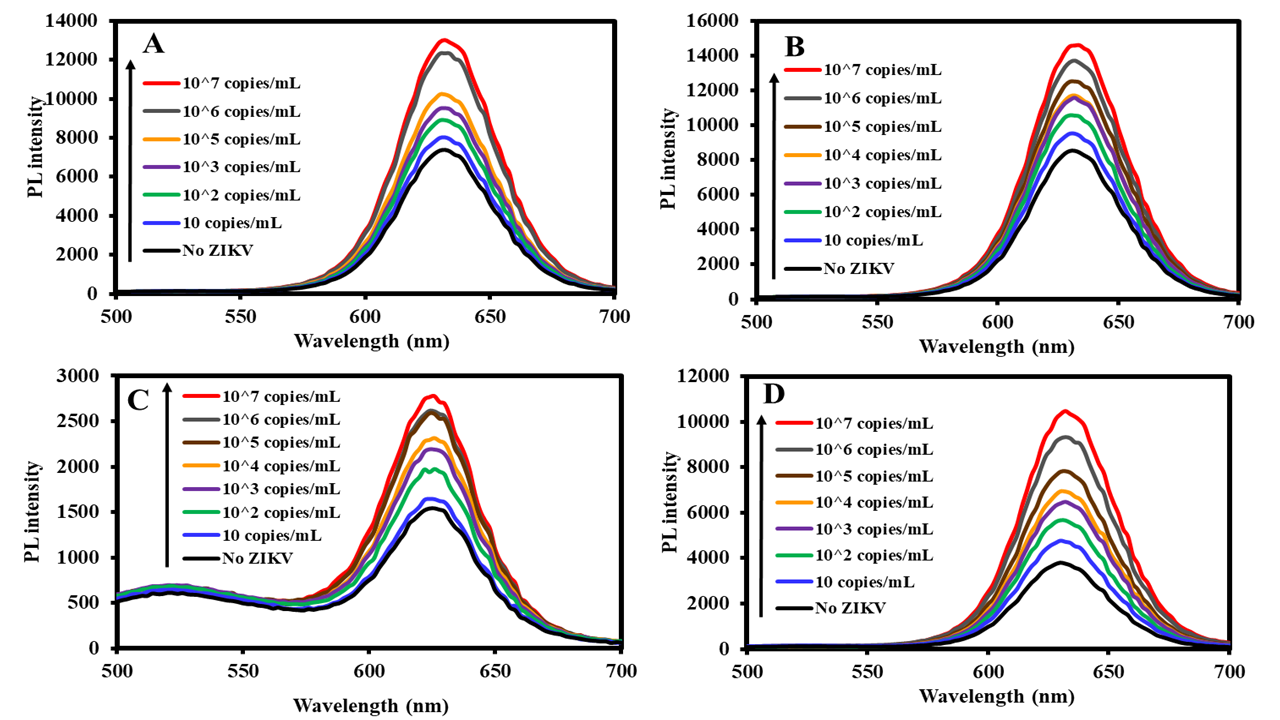

Supplement: S7 Fig — Fluorescence enhancement spectrum of the Ab-QDs as it correlates to the detected ZIKV using the LSPR signal amplifier of A) Ab-GSH-AuNPs, B) Ab-TGA-AuNPs, C) Ab-MPA-AuNPs, and D) Ab- L-cyst-AuNPs. (TIF) [file pone.0211517.s007.tif]

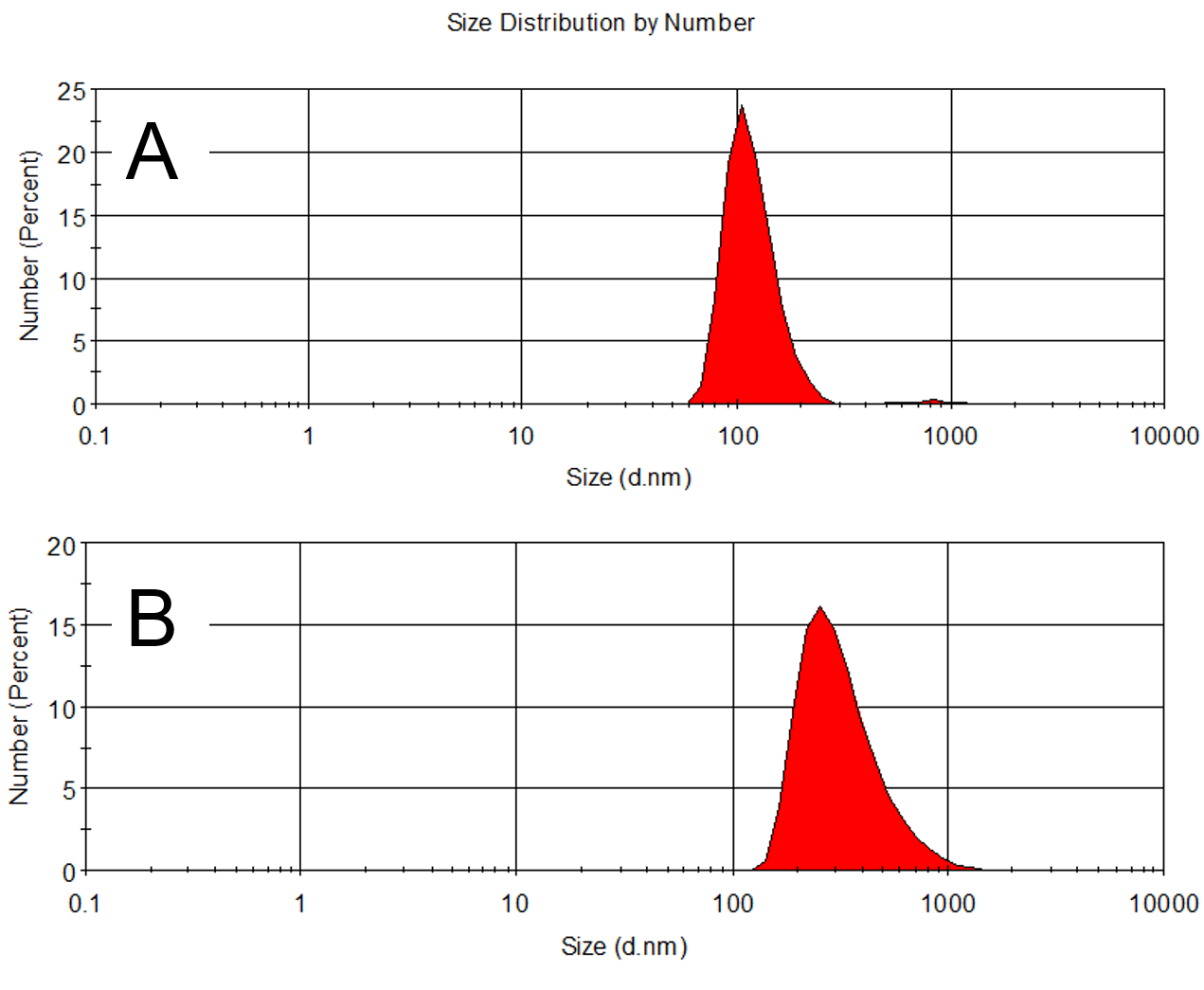

Supplement: S8 Fig — DLS hydrodynamic curves for A) QD-Ab-ZIKV-Ab-MPA-AuNPs and B) QD-Ab-ZIKV-Ab-L-cyst-AuNPs. Peaks of A, B and C are 38.6, 106.8 and 212.0 nm, respectively. (TIF) [file pone.0211517.s008.tif]

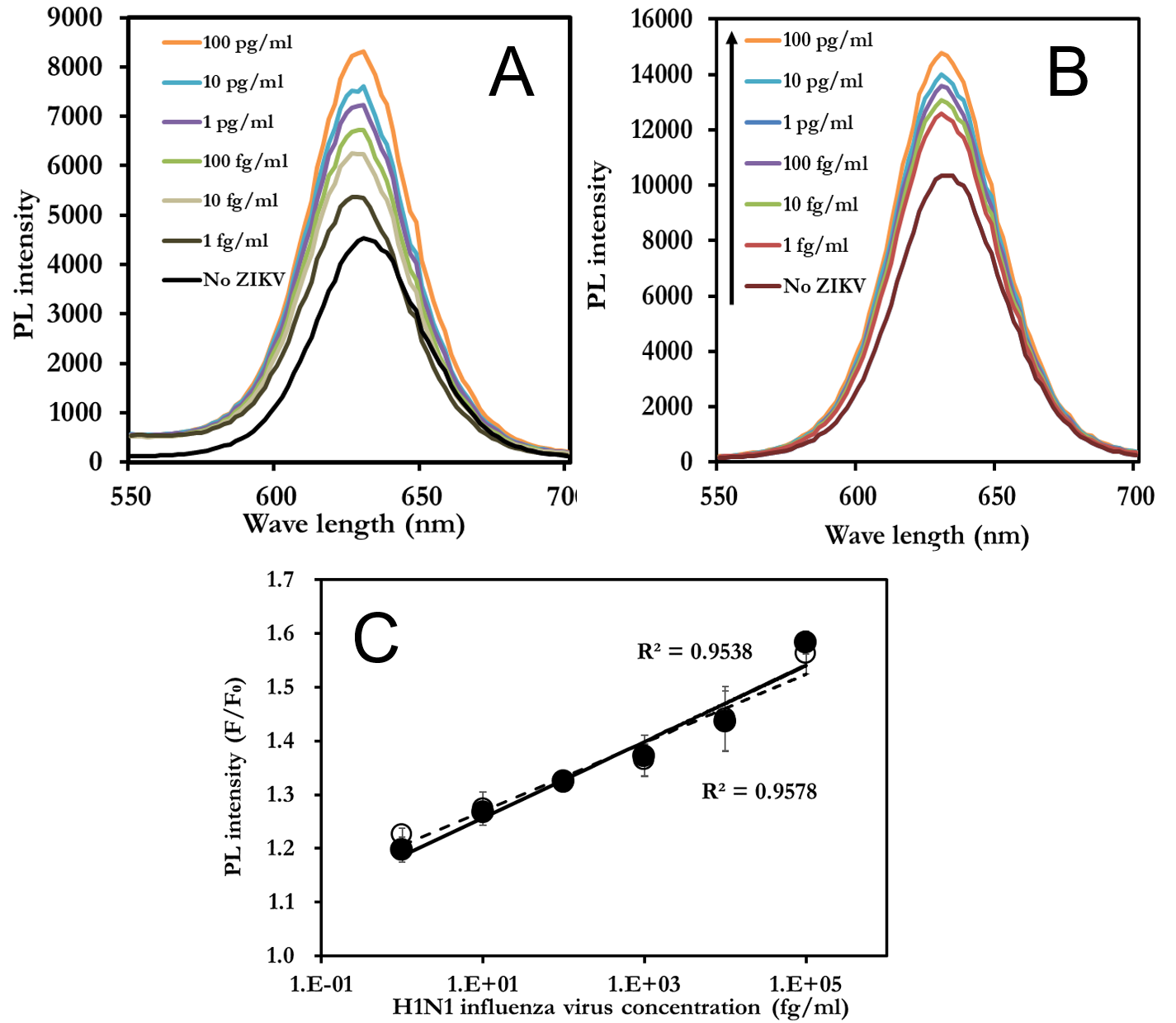

Supplement: S9 Fig — Influenza virus A (H1N1) was detected using the LSPR signal amplifier of Ab-MPA-AuNP in DI water (A), in human serum (B). Calibration curve (C) for Ab-MPA AuNP in DI water (〇) and in human serum (●). (TIF) [file pone.0211517.s009.tif]
